# Prevalence of Anemia and its Associated Factors among Chinese 9-, 12-, and 14-Year-Old Children: Results from 2014 Chinese National Survey on Students Constitution and Health

**DOI:** 10.3390/ijerph17051474

**Published:** 2020-02-25

**Authors:** Zhaogeng Yang, Yanhui Li, Peijin Hu, Jun Ma, Yi Song

**Affiliations:** Institute of Child and Adolescent Health & School of Public Health, Peking University, No. 38 Xueyuan Road, Haidian Dsictrict, Beijing 100191, China; yangzhaogeng@bjmu.edu.cn (Z.Y.); yanhuili@bjmu.edu.cn (Y.L.); peijinhu@bjmu.edu.cn (P.H.)

**Keywords:** anemia, nutrition, dietary intake, adolescents

## Abstract

**Background:** Anemia has been one of the main nutritional challenges around the world. Not enough attention has been paid to this issue in children and adolescents in China. In this study, we aimed to estimate the prevalence of anemia among 9-, 12-, and 14-year old Chinese children and investigate the associated factors of anemia. **Methods:** Data come from a cross-sectional survey conducted in 26 provinces and 4 municipalities in mainland China. A total of 48,537 children aged 9, 12, and 14 years old were included in data analyses. Anthropometric measurements were conducted to obtain information about height and weight. Capillary blood was collected from the fingertip, and hemoglobin concentration was tested by HemoCue201+. Information about sleep duration, daily consumption of eggs, milk, and breakfast were obtained from a self-administrated questionnaire. The mixed-effects logistic regression model was applied to estimate the association between selected variables and risk of anemia. **Results:** A total of 8.4% of participants were identified as being anemic; and the prevalence was higher in girls and rural children. Mixed-effects logistic regression analysis showed that children who were overweight, obese, and consumed eggs and milk every day had a lower risk of anemia. Spermarche, overweight/obesity, and having milk every day were associated with lower risk of anemia in boys, while menarche was found to be a risk factor and eating eggs every day to be a protective factor of anemia in girls. **Conclusions:** Anemia among 9-, 12-, and 14-year-old children is still high. Intervention programs of adding egg and milk into school daily diet might contribute to reducing anemia in Chinese school aged children, especially for those living in rural areas or girls with menarche.

## 1. Introduction 

Anemia is regarded as the most common micronutrient deficiency disorder around the world [1], which affects both developing and developed countries [2]. Hemoglobin is an iron containing oxygen that transports metalloprotein in the red blood cells [3], and it has been recognized that anemia results from the number and size of red blood cells or a hemoglobin concentration below the established cut-off value, with subsequent impairment in meeting the oxygen delivery demands of tissues [4]. According to estimates, anemia has affected approximately 25% of school-aged children (5–14 years old) globally [5]. Data from Global Burden of Disease 2013 [6] showed that iron deficiency anemia (IDA) was the leading cause of years lived with disability (YLDs) in children and adolescents, affecting 619 (95%UI: 618–621) million prevalent cases. South Asia is one of the regions with the highest prevalence of cases, and China has contributed the second largest number of cases (75.8 million) around the world [6]. 

The pathophysiology of anemia is diverse and often multifactorial, and possible causes include genetic mutations in hemoglobin genes, acute and chronic blood loss, inadequate nutrients intake, and infectious processes. Of them, iron deficiency is assumed to be the major one [7,8]. Previous studies have demonstrated that anemia was associated with adverse effects on individuals’ well-being, and if left untreated, could cause impaired tissue oxygen delivery and may lead to fatigue, lethargy, concentration difficulty, impaired physical capacity, poor work performance, and even mental disorders [9,10,11,12].

Although people at any time and at all stages of life may face the problem of anemia, children and pregnant women are deemed to be at greater risk than the standard population [7]. Over the past years, more attention has been focused on pregnant women and children under five, but there have been less studies concerning school aged children.

This study uses data from the Chinese National Surveys on Students Constitution and Health (CNSCCH) in 2014, which is, so far, the largest nationally representative sample of school aged children. The objectives of our study were: (1) to estimate the concentration of hemoglobin and prevalence of anemia in Chinese 9-, 12-, and 14-year-old children and compare the differences between sex, residence area, and age; and (2) to explore the potential associated factors with anemia.

## 2. Methods

### 2.1. Study Design 

Data was obtained from the 2014 cycle of the Chinese National Survey on Students Constitution and Health (CNSSCH), which is a joint nationwide investigation conducted every five years by the Ministries of Education, Health, Science, and Technology, the State Ethnic Affairs Commission, and the State Sports General Administration of China since 1985. Subjects were Han students from 26 mainland provinces and 4 municipalities, excluding Tibet (where the Han ethnicity is a minority), and a more detailed description of the study design and conduct can be assessed elsewhere [13]. Briefly, a multistage cluster sampling method was applied to determine participants. At first, three regions were randomly selected from each province, and several schools were randomly chosen from each region in 1985. From then on, the sampling framework has been kept uniform. In each school, sampling took place in classes randomly selected from each grade, and all selected children were invited to participate in this survey. All participants were enrolled in the later anthropometric measurements, such as height and weight. Additionally, 9-, 12-, and 14-year-old children completed capillary blood collection, hemoglobin concentration test, and self-administrated questionnaire. Informed consent was obtained from both children and their parents. Thus, a total of 48,537 participants were included in this study. All survey sites used the same protocol during the implementation process. The survey was approved by the Medical Research Ethics Committee of the Peking University Health Science Center (IRB00001052-18002).

### 2.2. Measurements

All measurements were conducted by trained investigators according to the standard protocol at all survey sites. During the anthropometric measurement process, subjects were asked to take off their coat, shoes, and wear only light clothing. Height was measured to the nearest 0.1 cm using a stadiometer (model TZG, Jiangyin Hongya Science and Education Equipment Co., Ltd.), and weight was recorded to the nearest 0.1 kg with a scale (model RGT-140, Wuxi Weighing Apparatus Factory Co., Ltd.). A 5% sample measured height and weight twice every day for quality control. If any part of readings exceeded the maximum acceptable difference for a given variable (e.g., 0.1 kg for weight, 0.5 cm for height), both records were measured once again independently and a second and, if necessary, third set of measurements were recorded. The final record was calculated by an average of the readings, both the stadiometer and scale were calibrated before use, and similar instruments were used in measurement at all survey sites. BMI was calculated as the weight (kg) divided by the square of the height (m^2^), and nutritional status was divided into thinness, normal BMI, overweight, and obese according to the gender- and age-specific Z-score for 7- to 18-year-old children developed by the WHO [14]. Thinness was defined as ≤ -2SD, normal BMI was defined as > -2SDa but ≤ 1SD, overweight was defined as > 1SD but ≤ 2SD, and obesity was defined as > 2SD. 

The capillary blood sample was collected from the fingertip after discarding the first drop. Hemoglobin concentration was measured by HemoCue201+ (Origin: Sweden, Model: HemoCue201 +, Manufacturer: HemoCue AB), and data collection was supervised daily by trained field supervisors. Anemia was diagnosed as <115 g/L for 9-year-old children and <120 g/L for 12- and 14-year-old children after corrected by altitude as proposed by the WHO criteria [15]. 

### 2.3. Questionnaires

Self-administrated structured questionnaires were applied in children to obtain information about age, spermarche (in boys)/menarche (in girls), sleep duration, and dietary behaviors, such as eating eggs, drinking milk, and having breakfast. Subjects were asked to report the average sleep duration over the past year by selecting from 6 options: (1) < 6 h; (2) 6 ~7 h; (3) 7~8 h; (4) 8~9 h; (5) 9~10 h and ≥10 h. Enough sleep duration was defined as ≥9 h for 9- and 12-year-old and ≥ 8 h for 14-year-old students according to recommendation of the American Academy of Sleep Medicine [16]. Children were also asked to report their frequency of having breakfast, eating eggs, and having milk by selecting from 4 options: (1) Never; (2) 1~2 times per week; (3) 3~4 times per week; and (4) every day. Ideal dietary intake was defined as every day.

### 2.4. Statistical Analysis

Continuous variables were characterized as mean ± standard deviation, and categorical variables were characterized by frequencies and percentages. One-way ANOVA was used to compare the distribution of nutritional status between boys and girls, independent sample Student’s *t*-test was used to compare the difference of hemoglobin concentration between sex and residence subgroup participants, and Chi-square test was used to compare the prevalence of anemia among children with different ages and residence areas. Considering that the multistage sampling method was used in this study and children from the same region and same school were more likely to have similar characteristics, the mixed-effects logistic regression model was used to examine the relationship between anemia and covariates by controlling the cluster effects. All analyses were conducted with univariable and multivariable models, respectively. 

At first, we use a bivariate model to assess association between anemia and selected variables, including nutritional status, sleep duration, eating eggs, drinking milk, having breakfast, and spermarche/menarche. Second, variables that were found significant in the bivariate regression models were then included in the mixed-effects multivariate logistic regression analyses to yield the final model. All analyses were performed using R software (version 3.4.0) and related packages “lme4” [17], and a two-sided *P* value of < 0.05 was considered statistically significant.

## 3. Results 

### 3.1. Descriptive Characteristics of the Study Population

The descriptive characteristics of the analyzed population are presented in Table 1. A total of 48,537 (24,235 boys and 24,464 living in urban area) students were included in this study. Of them, 3.9% were classified as thinness, 16.0% were overweight, and 7.4% were obese, respectively. The percentage of enough sleep duration, eating eggs every day, having breakfast every day, and having milk every day were 28.0%, 14.9%, 76.9%, and 38.0%, respectively. Boys were observed to have a higher prevalence of overweight and obesity categories and a higher percentage of enough sleep duration, eating eggs every day, and having milk every day when compared with girls (*p* < 0.001). 

### 3.2. Hemoglobin Concentration and Prevalence of Anemia

Table 2 presents the hemoglobin concentration among 9-, 12-, and 14-year-old children. The mean hemoglobin(Hb) concentration was 135.2 ± 12.8 g/L in all participants, it was lower in girls (132.4 ± 11.6 g/L) than boys (138.0 ± 13.3 g/L), and lower in rural children (134.7 ± 12.6 g/L) than urban peers (135.6 ± 13.0 g/L) (*p* < 0.001). The prevalence of anemia was 8.4% in all participants (Table 3), and the prevalence in girls was significantly higher than that of boys among all participants and residence-stratified and age-stratified subgroups (*p* < 0.001). In addition, the prevalence of anemia was higher in rural children (8.9%) than urban children (8.0%) (*p* < 0.001); however, when analyzed stratified by sex and age, the difference was still significant only in 12- and 14-year-old girls.

### 3.3. Factors Associated with Anemia

Potential associated factors were analyzed in a mixed-effects model. Univariate models (Table 4) showed that higher age [12 years old (OR:1.48; 95% CI: 1.30–1.69), 14 years old (OR:1.35; 95%CI:1.16–1.56)] and spermarche/menarche (OR:1.53; 95% CI:1.40–1.67) were associated with increased risk of anemia. On the contrary, those who were overweight (OR:0.88; 95%CI:0.80–0.97), obese (OR:0.64; 95%CI:0.55–0.75), eating eggs every day (OR:0.88; 95%CI: 0.79–0.98), and having milk every day (OR:0.88; 95%CI:0.82–0.95) were observed to have a lower risk of anemia. Multivariate models showed that those who were overweight (OR:0.84; 95%CI:0.75–0.94), obese (OR:0.65; 95%CI: 0.53–0.79), eating eggs every day (OR:0.89; 95%CI: 0.79–0.98), and having milk every day (OR:0.90; 95%CI: 0.82–0.97) had a lower risk of anemia. In contrast, children with spermarche/menarche were more vulnerable to be anemic (OR:1.60; 95%CI: 1.45–1.77), and there were insignificant associations between sleep duration and having breakfast every day and risk of anemia. When further analyzed by sex, a lower risk of anemia was observed in boys with higher age, obesity, having milk every day, having breakfast every day, and spermarche. While, for girls, eating eggs every day was a protective factor, but higher age and menarche were risk factors for anemia (Table 5).

## 4. Discussion

Anemia is one of the most common nutritional problems affecting adolescents from both developing and developed countries. In this study, we observed a prevalence of 8.4% for anemia in Chinese 9-, 12-, and 14-year-old children, and a higher prevalence was observed in girls and rural children. We also found that eating eggs every day and having milk every day were associated with lower risk of anemia.

As expected, anemia in Chinese 9-, 12-, and 14-year-old children and adolescents represented a mild public health problem according to the WHO criteria [18]. The prevalence of anemia in the present study was lower than the2010 CNSSCH survey (9.9%), and a marked decreasing trend was observed when compared to the CNSSCH surveys conducted between 1995 and 2010 [19]. This was parallel with the nutritional improvement over the past years. We also observed a higher prevalence than the results from the Chinese National Nutritional and Health Survey (CNNHS) program study conducted in 2010–2012 (4.3% for 9–11 years, and 7.2 for 12–14 years) [20], and this may result from the difference in sample and blood collection methods applied in those surveys. When compared with results from other countries, the prevalence of anemia in the present study was lower than that from other low- and middle-income countries, such as India (21.4%) [21], Mexico (12%) [5], and Ethiopia (33.9%) [22], but higher than some developed regions, such as the United States (9% in 12–15 years old) [5], Colombia (4%) [5], and Japan (3.6% in boys and 2.5% in girls) [23].

In the present study, we found that girls and rural children had lower hemoglobin concentration and a higher prevalence of anemia, which was consistent with a study conducted in India [24]. One possible reason may be due to the difference in pubertal development between boys and girls, as girls lose blood during menstruation and thus have a higher requirement for hemoglobin and iron [20,25].

Understanding factors correlated with anemia can provide more insight to the measures that can be applied to combat anemia. In the present study, we found that overweight and obese children were less likely to have anemia, which was consistent with a study conducted by Hoang [26], although other studies had detected both positive [27,28] and insignificant [29,30] correlations between anemia and BMI. Hoang et al. [26] found that overweight/obese children may have had a higher hemoglobin concentration compared to normal weight or underweight children for some reasons that have not yet been explored. Furthermore, we also found that children who consume eggs and milk every day had a lower risk of anemia. Tibambuya BA et al. [31] also found that consumption of egg three or more times in a week could independently reduce the odds of anemia in pregnant women. Egg and cow’s milk are ubiquitous globally and are known as top contributors for all nutrients as well as an exceptional protein source with a large range of micronutrients [32,33,34,35,36], and more importantly, egg is a fairly good source of iron [37] and milk is a good source of protein. Eggs and milk were widely used in previous nutritional intervention programs, such as the Lulin project [34], a randomized controlled trial conducted among young undernourished children in the Ecuadorian Andes, which showed dramatic effects on growth and stunting reduction via consuming eggs for just six months.

Although egg and milk are considered to be low cost but high benefit foods, they are not widely consumed among Chinese people, and according to estimates, mean national milk intake in China was 23.4 g/day in 2015 [38], which was the lowest among 185 investigated countries and much lower than the global average (95.0 g/day, 95% uncertainty interval: 70.1–134.7 g/day). Furthermore, mean national egg consumption in China was also under the global average level [38]. Over the past years, the Chinese government has taken some measures to improve nutritional status of school aged children, especially in rural areas. In 2011, the “Free-Lunch for Children” project [39], a charitable program aimed at providing free and nutritious lunch for rural areas students, was launched in parts of regions in China. In the same year, the Chinese government launched the Nutrition Improvement Program for Rural Compulsory Students (NIPRCES) project [40], aimed at providing basic nutrients for improving the nutritional status of poor rural pupils, and egg and milk were included in the implementation plan and served in the school meals. About 23 million rural compulsory students were covered by NIPRCES project in 2015, but according to Zhang’s estimate [40], the portion size and amount were hard to meet the requirements of school children, and egg and milk consumption needed a wider range of promotion. 

The major limitation of this study was cross-sectional design, which weakened the capacity in revealing the temporal sequence between the factors and anemia. Furthermore, information about sleep duration and eating habits were obtained from self-administrated questionnaires rather than a validated scale, which may lead to poor precision and incomprehensive measurements, and we also did not collect information about household composition, social economic status, and detailed dietary intake, which were regarded as potential confounding factors of anemia. Moreover, participants of this study included only 9-, 12-, and 14-year-old children, who cannot represent the whole adolescent population. Further studies with reasonable and scientific design are needed to prove the results in the present study.

## 5. Conclusions

Overall, anemia prevalence was still relatively high among Chinese 9-, 12-, and 14-year-old children, even though it has been improved over the past years. Gender and urban–rural disparities exist, e.g., girls and rural children were more likely to suffer from anemia. Children with overweight/obesity status, spermarche, eating eggs every day, and having milk every day were associated with a lower risk of anemia, and menarche in girls was a risk factor for anemia. Intervention programs of adding egg and milk into school daily diet might contribute to reducing anemia in Chinese school aged children, especially for those living in rural areas or girls with menarche. 

## Figures and Tables

**Table 1:** Descriptive characteristics of the population included in this study.

Variables	Total (n = 48537)	Boys (n = 24235)	Girls (n = 24302)	*p*-value
Area of residence, %				
urban	24464(50.4)	12193(50.3)	12271(50.5)	0.722 ^#^
rural	24057(49.6)	12029(49.7)	12028(49.5)	
Age, years				
9	13381(27.6)	6632(27.4)	6749(27.8)	0.617 ^#^
12	17531(36.1)	8782(36.3)	8749(36.0)	
14	17609(36.3)	8808(36.4)	8801(36.2)	
Nutritional status				
Normal weight	35250(72.6)	16016(66.1)	19234(79.2)	<0.001 *
Thinness	1891(3.9)	945(3.9)	946(3.9)	
Overweight	7780(16.0)	4504(18.6)	3276(13.5)	
Obesity	3600(7.4)	2757(11.4)	843(3.5)	
Sleeping duration				
Not enough	34957(72.0)	17041(70.4)	17916(73.7)	<0.001 ^#^
Enough	13564(28.0)	7181(29.6)	6383(26.3)	
Eating eggs every day				
Yes	7212(14.9)	3842(15.9)	3370(13.9)	<0.001 ^#^
No	41309(85.1)	20380(84.1)	20929(86.1)	
Having milk every day				
Yes	18450(38.0)	9634(39.8)	8816(36.3)	<0.001 ^#^
No	30071(62.0)	14588(60.2)	15483(63.7)	
Having breakfast every day				
Yes	37293(76.9)	18553(76.6)	18740(77.1)	0.166 ^#^
No	11227(23.1)	5677(23.4)	5562 (22.9)	
Spermarche/menarche				
Yes	20287(53.3)	6659(44.0)	13628(59.5)	<0.001 ^#^
No	17749(46.7)	8481(56.0)	9268(40.5)	

Notes: # indicates the statistical test was conducted by Chi-square test, * indicates the statistical test was conducted by one-way ANOVA.

**Table 2:** The mean hemoglobin concentration in 9-, 12-, and 14-year-old children, by sex and residence area.

Age	Residence	Total	Boys	Girls	*p*-value *
9	Total	131.0 ± 10.7	131.5 ± 10.7	130.6 ± 10.7	<0.001
	Urban	131.2 ± 10.8	131.6 ± 10.8	130.8 ± 10.7	0.007
	Rural	130.9 ± 10.6	131.4 ± 10.6	130.4 ± 10.6	<0.01
	*p*-value ^#^	0.015	0.521	0.107	
12	Total	135.0 ± 12.1	136.6 ± 12.3	133.4 ± 11.8	<0.001
	Urban	135.5 ± 12.2	137.0 ± 12.3	134.0 ± 11.8	<0.001
	Rural	134.5 ± 12.0	136.3 ± 12.2	132.8 ± 11.6	<0.001
	*p*-value ^#^	<0.001	0.008	<0.001	
14	Total	138.4 ± 14.0	144.2 ± 13.3	132.7 ± 12.1	<0.001
	Urban	139.0 ± 14.2	145.1 ± 13.5	133.0 ± 12.1	<0.001
	Rural	137.8 ± 13.8	143.4 ± 13.1	132.3 ± 12.1	<0.001
	*p*-value ^#^	<0.001	<0.001	0.004	
Total	Total	135.2 ± 12.8	138.0 ± 13.3	132.4 ± 11.6	<0.001
	Urban	135.6 ± 13.0	138.4 ± 13.6	132.8 ± 11.7	<0.001
	Rural	134.7 ± 12.6	137.6 ± 13.1	131.9 ± 11.6	<0.001
	*p*-value ^#^	<0.001	<0.001	<0.001	

Notes: * indicates the *p*-value was obtained from Chi-square test between boys and girls; # indicates the *p*-value was obtained from Chi-square test between urban and rural children.

**Table 3:** Prevalence of anemia in 9-, 12-, and 14-year-old age children, by sex and residence area.

Age	Residence	Total	Boys	Girls	*p*-value *
9	Total	6.8	6.4	7.1	0.155
	Urban	6.6	6.4	6.8	0.477
	Rural	6.9	6.5	7.4	0.193
	*p*-value ^#^	0.465	0.837	0.413	
12	Total	9.6	7.7	11.5	<0.001
	Urban	8.8	7.4	10.3	<0.001
	Rural	10.3	7.9	12.8	<0.001
	*p*-value ^#^	0.001	0.364	<0.001	
14	Total	8.5	3.5	13.5	<0.001
	Urban	8.1	3.7	12.6	<0.001
	Rural	8.9	3.4	14.4	<0.001
	*p*-value ^#^	0.076	0.493	0.016	
Total	Total	8.4	5.8	11.0	<0.001
	Urban	8.0	5.8	10.2	<0.001
	Rural	8.9	5.9	11.9	<0.001
	*p*-value ^#^	<0.001	0.668	<0.001	

Notes: * means comparison between boys and girls. ^#^ means comparison between urban and rural children.

**Table 4:** Association between anemia and its risk factors in univariate models.

Variables	Total		Boys		Girls	
	OR (95% CI)	*p*-value	OR (95% CI)	*p*-value	OR (95% CI)	*p*-value
Age						
9	Reference		Reference		Reference	
12	1.48(1.30,1.69)	<0.001	1.24(1.03,1.48)	0.022	1.73(1.50,2.09)	<0.001
14	1.35(1.16,1.56)	<0.001	0.51(0.41,0.64)	<0.001	2.17(1.79,2.2.60)	<0.001
Nutritional status						
Normal weight	Reference					
Thinness	0.92(0.78,1.10)	0.363	1.19(0.91,1.57)	0.200	0.87(0.69,1.10)	0.238
Overweight	0.88(0.80,0.97)	0.007	0.99(0.85,1.15)	0.896	0.93(0.82,1.06)	0.309
Obesity	0.64(0.55,0.75)	<0.001	0.74(0.59,0.91)	0.005	0.85(0.72, 0.97)	0.021
Sleep duration						
Not Enough	Reference					
Enough	1.00(0.92,1.08)	0.902	1.07(0.94,1.22)	0.320	1.02(0.91,1.13)	0.769
Eating eggs every day						
No	Reference					
Yes	0.88(0.79,0.98)	0.018	1.02(0.87,1.21)	0.798	0.83(0.72,0.95)	0.009
Having milk every day						
No	Reference					
Yes	0.88(0.82,0.95)	0.001	0.90(0.79,1.01)	0.084	0.90(0.82,0.99)	0.041
Having breakfast every day						
No	Reference					
Yes	0.93(0.86,1.01)	0.074	0.99(0.86,1.14)	0.908	0.88(0.80,0.97)	0.020
Spermarche/menarche						
No	Reference					
Yes	1.53(1.40,1.67)	<0.001	0.54(0.45,0.65)	<0.001	1.56(1.56,1.56)	<0.001

Notes: 95% confidence interval and *p*-value were generated from the multivariable mixed-effects logistic regression model. Abbreviations: OR = odds ratio, CI = confidence interval.

**Table 5:** Association between anemia and its risk factors in multivariate models.

Variables	Total		Boys		Girls	
	OR (95% CI)	*p* value	OR (95% CI)	*p* value	OR (95% CI)	*p*-value
Age						
9	Reference					
12	1.39(1.16,1.66)	<0.001	0.56(0.18,1.73)	0.313	2.06(1.66,2.56)	<0.001
14	1.06(0.87,1.30)	0.544	0.27(0.08,0.83)	0.022	2.57(2.01,3.28)	<0.001
Nutritional status						
Normal weight	Reference					
Thinness	0.98(0.81,1.19)	0.846	1.25(0.90,1.74)	0.179	0.91(0.71,1.17)	0.471
Overweight	0.84(0.75,0.94)	0.001	0.83(0.70,0.97)	0.022	0.95(0.83,1.08)	0.445
Obesity	0.65(0.53,0.79)	<0.001	0.61(0.44,0.86)	0.005	0.85(0.72,0.97)	0.013
Sleep duration						
Not Enough	Reference					
Enough	1.05(0.96,1.15)	0.312	1.20(0.98,1.43)	0.201	1.06(0.94,1.19)	0.333
Eating eggs every day						
No	Reference					
Yes	0.89(0.79,0.98)	0.042	1.08(0.85,1.37)	0.537	0.84(0.72,0.97)	0.016
Having milk every day						
No	Reference					
Yes	0.90(0.82,0.97)	0.010	0.83(0.70,0.98)	0.031	0.96(0.86,1.06)	0.427
Having breakfast every day						
No	Reference					
Yes	0.96(0.88,1.06)	0.432	0.88(0.78,0.99)	0.046	0.96(0.86,1.07)	0.415
Spermarche/menarche						
No	Reference					
Yes	1.60(1.45,1.77)	<0.001	0.79(0.64,0.97)	0.003	1.14(0.99,1.31)	0.077

Notes: 95% confidence interval and *p*-value were generated from the multivariable mixed-effects logistic regression model. Abbreviations: OR = odds ratio, CI = confidence interval.
